# Risk factors for complicated grief among family members bereaved in intensive care unit settings: A systematic review

**DOI:** 10.1371/journal.pone.0264971

**Published:** 2022-03-10

**Authors:** Emma A. M. Sanderson, Sally Humphreys, Fiona Walker, Daniel Harris, Emma Carduff, Joanne McPeake, Kirsty Boyd, Natalie Pattison, Nazir I. Lone

**Affiliations:** 1 Usher Institute, University of Edinburgh, Edinburgh, United Kingdom; 2 University of Hertfordshire, Hatfield, United Kingdom; 3 NHS Lothian, Edinburgh, United Kingdom; 4 Cambridge University Hospitals NHS Foundation Trust, Cambridge, United Kingdom; 5 Marie Curie Hospice, Glasgow, United Kingdom; 6 University of Glasgow, Glasgow, United Kingdom; 7 East and North Herts NHS Trust, Stevenage, Hertfordshire, United Kingdom; Universitat d’Alacante, SPAIN

## Abstract

**Background:**

Families of intensive care unit (ICU) decedents are at increased risk of experiencing complicated grief. However, factors associated with complicated grief in ICU and bereavement needs assessment are not available routinely. We aimed to conduct a systematic review identifying risk factors associated with complicated grief among family members of ICU decedents.

**Materials and methods:**

MEDLINE, EMBASE, CINAHL, PsycINFO, the Cochrane Library and Web of Science were searched to identify relevant articles. Observational studies and randomised and non-randomised controlled trials were included. Studies were screened and quality appraised in duplicate. Risk of bias was assessed using Newcastle-Ottawa Scale. A narrative synthesis was undertaken.

**Results:**

Seven studies conducted across three continents were eligible. Four studies were of high quality. 61 risk factors were investigated across the studies. Factors associated with a decreased risk of complicated grief included age, patient declining treatment and involvement in decision-making. Factors associated with increased risk included living alone, partner, dying while intubated, problematic communication, and not having the opportunity to say goodbye.

**Conclusion:**

This systematic review has identified risk factors which may help identify family members at increased risk of complicated grief. Many of the studies has small sample sizes increasing the risk of erroneously reporting no effect due to type II error. Some factors are specific to the ICU setting and are potentially modifiable. Bereavement services tailored to the needs of bereaved family members in ICU settings are required. (PROSPERO registration ID 209503)

## Introduction

Most people will experience bereavement during their lifetime. For many, the period following death can be disruptive and time is needed to adjust and accept the death of a loved one. Most individuals adapt to their loss and can integrate back into their life, often within a few months after the death [1]. However, a proportion of individuals experience prolonged, intense grieving that affects physical and mental health, and impacts on social and emotional wellbeing. This response is referred to as complicated grief [2]. Features may include guilt, anger, denial, emotional numbness or difficulty in engaging with activities [2]. Complicated grief is increasingly referred to as “prolonged grief disorder” and this term is now included in both the 11th edition of the International Classification of Diseases-11 and the 5th edition of the Diagnostic and Statistical Manual of Mental Disorders [3].

The proportion of individuals who experience complicated grief following a bereavement has been estimated to be between 5 and 10% in the general population [4]. In contrast, studies have shown that there is a much higher prevalence in family members bereaved in intensive care unit (ICU) settings, with studies estimating a prevalence of 46%-52% [5, 6]. Risk factors for complicated grief have been identified in non-ICU settings, which have led to the development of bereavement risk assessment screening tools for hospice and community settings [7]. However, given the highly technical environment and the frequency of sudden deaths in ICU compared to the wider hospital or community settings, it is possible that individual factors which are specific to the ICU context have not been considered in previous work. Furthermore, the COVID-19 pandemic has highlighted the unique challenges of supporting family members during and after end-of-life care in ICUs [8, 9].

Several reviews have been conducted previously to evaluate risk factors for complicated grief in the general population [10, 11], and specifically in family caregivers [12, 13]. However, there are no published systematic reviews specifically investigating risk factors for complicated grief in the ICU context. Therefore, we aimed to systematically review published studies investigating factors associated with complicated grief among family members of patients dying in ICU (ICU decedents).

## Methods

The Preferred Reporting Items for Systematic Review and Meta-Analysis Protocols (PRISMA-P) [14] was used to develop the protocol for this systematic review and was then registered (PROSPERO ID: 209503). The PRISMA checklist was followed to conduct and report the review [15]. Studies included in the review were limited to those in which study participants were adult family members of patients admitted to adult ICUs. No limitations were in place for publication dates. Cohort studies, case-control studies, cross-sectional studies and randomised and non-randomised controlled trials were eligible to be included. Editorials, case-reports, case-series, conference proceedings and qualitative research were excluded. Publications with only abstracts were also excluded. In addition, studies that did not provide a quantitative measure of association with the outcome were excluded. Furthermore, studies which investigated families of patients admitted to paediatric or neonatal critical settings were excluded. Due to a lack of translation resource, the review was limited to studies published in English.

### Databases and search strategy

Search strategies were developed with the help of a medical librarian and the following databases were searched on 05/01/2022: MEDLINE, EMBASE, CINAHL, PsycINFO, the Cochrane Library and Web of Science. In addition, reference lists of the identified papers and review articles were searched to identify any further studies. We used a combination of controlled vocabulary and non-controlled terms to capture the main concepts in search strategies: “intensive care”, “bereavement”, “risk factors” and “complicated grief” (see S1 Table for complete search strategy).

### Data management and extraction

The results from the search were imported into a reference management software package and duplicates were removed. Abstracts were independently screened by two reviewers and full text of papers were retrieved and independently assessed in duplicate. Reviewers used eligibility criteria determined *a priori*. Disagreements were resolved by involving a third review author.

The following data were extracted by a single reviewer and tabulated from eligible articles: publication information (study author, year and years conducted), study characteristics (including study design and statistical methodology), participant characteristics (population, sample size and relationship to deceased), risk factors (number reported or assessed, when they were ascertained and how they were ascertained) and outcome measures (definition, measurement tool, proportion of individuals with outcome and when outcome was assessed). The result of interest was the association between potential risk factors and complicated grief. Where possible odds ratios, risk ratios or hazard ratios were extracted for both univariable and multivariable analyses along with the relevant 95% confidence intervals. If this was not available, another relevant measure of association was extracted with its corresponding p-value. Data were extracted for all time points where results were reported.

### Quality assessment, risk of bias and data synthesis

The Newcastle-Ottawa Scale (NOS) [16] was used to assess the risk of bias for observational studies. It was conducted independently by two reviewers, with the results synthesised together by a third reviewer. Any disagreements were resolved using the third reviewer. Results were tabulated and a narrative synthesis was conducted due to great variability in the risk factors assessed and measures of association, thus precluding a meta-analysis.

## Results

### Study selection and characteristics

**Fig 1:**
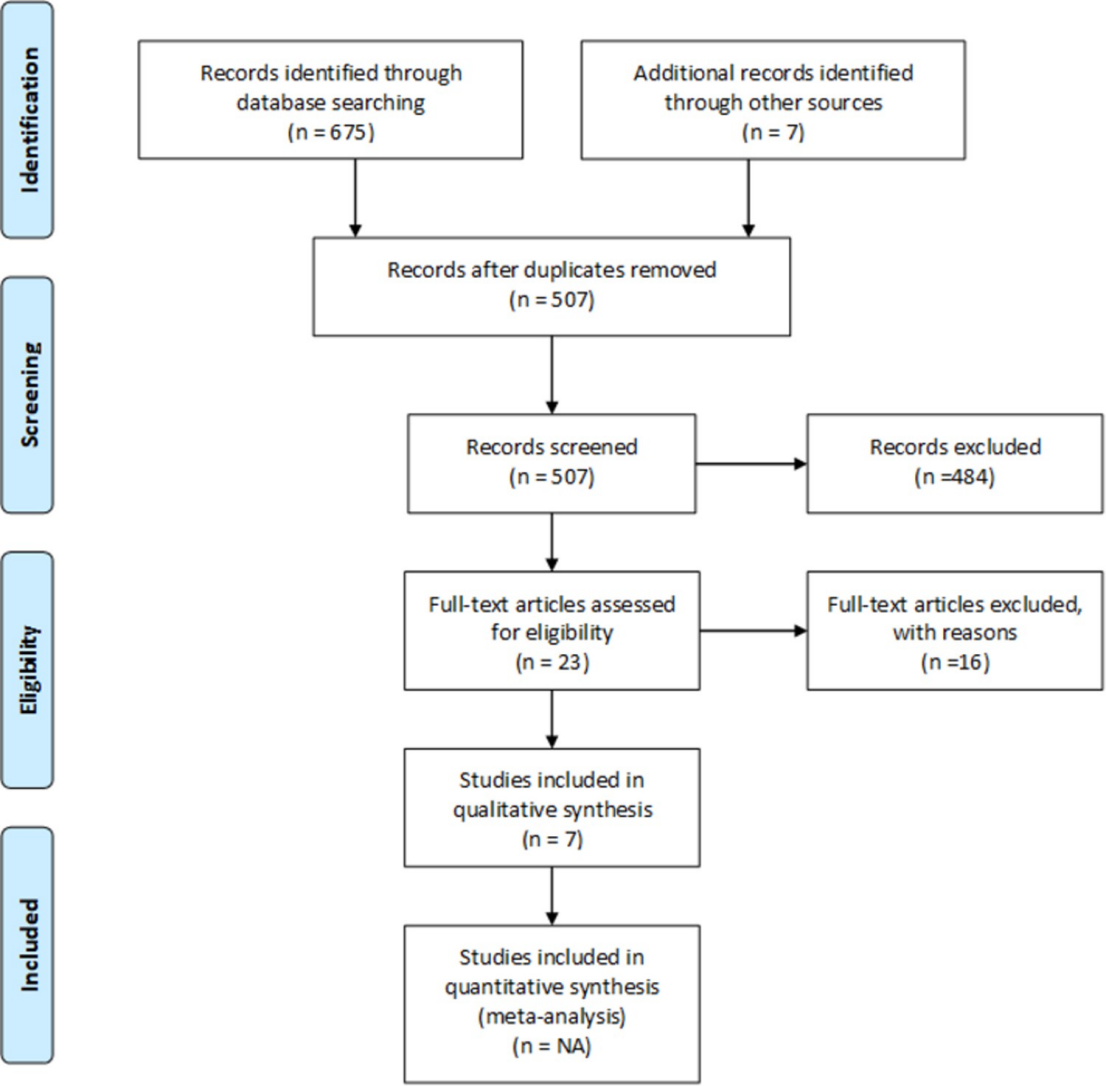
PRISMA flow diagram indicating the selection process for articles identifying risk factors of complicated grief for family members of ICU decedents.

Fig 1 shows the study selection process. Of the 574 articles identified, seven were eligible to be included in this review [6, 17–22] The main characteristics of the studies are described in Tables 1 and 2. The studies were conducted between 2005 and 2015, four were conducted in France and the remaining three in Australia, Canada and the USA. Almost all studies were observational in nature, apart from one interventional study [18]. Most relatives included were children or a spouse/partner of the patient. The Inventory of Complicated Grief (ICG) or ICG-Revised was used in six studies and the Core Bereavement Items questionnaire for one [17]. The ICG is a 19-item questionnaire with first-person statements scored using a Likert scale. A score greater than 25 suggests complicated grief is present [23]. The Core Bereavement Items questionnaire consists of 17 questions measuring the intensity of bereavement reactions by assessing ‘core’ bereavement experiences [24].

**Table 1:** Publication data, study design information and details concerning the population for the 7 studies selected which assess potential risk factors for complicated grief.

Author	Year	Years Conducted	Country	Study Design; study purpose	Population; No hospitals/ICUs included	Sample Size	Relationship to patient (%)
**Buckley et al. **	2015	2005–2008	Australia	Prospective cohort; describe relationship between nature of death and bereavement intensity and examine modifying effects of coping responses	Bereaved relatives of critical care unit patients who were also participating in the Cardiovascular Health in Bereavement study; 5 hospitals	78	Spouse/Partner 92; Parent 8
**Downar et al. **	2018	NR	Canada	Sequential explanatory design; measure burden and predictors of severe grief reactions, psychiatric illness and social distress	Bereaved family members of ICU patient; 9 hospitals	106	Spouse/Partner 49; Child 26; Sibling 8; Parent 8; Other 8
**Kentish- Barnes et al.**	2015	2011–2013	France	Prospective observational; determine prevalence and risk factors for CG	Relatives of patients who’d died after at least 48 hours in ICU and had visited ICU at least once; 41 ICUs	475	NR
**Kentish-Barnes et al. **	2016	2011–2013	France	Mixed methodology; creating a new questionnaire (CAESAR) to assess relatives experience of dying and death in ICU	Relatives of patients who’d died after at least 48 hours in ICU and had visited ICU at least once; 41 ICUs	475	Spouse 33.9; Adult child 45.5; Parent 3.1; Sibling 9; Other 8.5
**Kentish-Barnes et al. **	2017	2014–2015	France	RCT; assessing effect of condolence letter	Family member of patient who had stayed in ICU at least 2 days prior to death and had visited at least once; 22 ICUs	242 (123 letter/119 control)	Adult child 40; Spouse/Partner 35.6; Other 24.4
**Robert et al. **	2017	2013–2014	France	Prospective observational; comparing IE to TW	Critically ill adults with decision to withdraw mechanical ventilation and main adult relative enrolled who had been in ICU at least 48 hours; 43 ICUs	190 IE /212 TW	IE: Partner 37.6; Adult Child 46; Parent 1.6; Other 14.8
							TW: Partner 31.1; Adult Child 45.8; Parent 4.2; Other 18.9
**Trevick and Lord **	2017	2014–2015	USA	Prospective cohort; investigate psychological outcomes of family and decision makers in neuro-ICU	Family members of severely ill neuro ICU patients where they met criteria for palliative care consult; 1 medical centre	41	Spouse 13.3; Parent 6.7; Child 36.7; Sibling 16.7; Niece/Nephew 3.3; Cousin 3.3; Other 20

Abbreviations: NR: Not reported; IE: Immediate extubation; TW: Terminal weaning; CG: Complicated grief; RCT: Randomised-controlled trial; ICU: Intensive care unit

**Table 2:** Details concerning outcome measurement, risk factor measurement and method of association between the two for the articles investigating potential risk factors for complicated grief.

Author	Timing of Risk Factor Measurement	Outcome	Method of outcome ascertainment	Definition of outcome	Time of outcome measurement (for CG)	Number with complicated grief outcome (%)	No risk factors assessed/reported	Method of ascertaining risk factors	Method of Association; selection process in model
**Buckley 2015**	Within 2 weeks bereavement; Coping determinants 3 and 6 months	Intensity of bereavement reaction	CBI-17	Not given	3 and 6 months	NA	22	Nature of death questionnaire and Brief COPE inventory	Forced entry linear regression; forced entry with variables p<0.1 from univariate analysis included
**Downar 2018**	3 months after bereavement	Severe grief reaction	ICG-revised	CG, PGD, PCBRD or total ICG score> 25	3 months after initial questionnaire (administered 3–5 months after death)	16 (19)	11	Questionnaire	Univariate linear Regression and kappa statistic; none
**Kentish- Barnes 2015**	21 days after death	Primary: CG Secondary: IES-r and HADS scores	ICG	ICG>25	6 and 12 months	6 months: 147 (52.1); 12 months: 113 (53)	19	Telephone interview with questionnaire; electronic case-record form completed by ICU staff	Mixed multivariate logistic regression; stepwise selection (where p<0.020 in univariate)
**Kentish-Barnes 2016**	21 days after death	CG, PTSD, anxiety and depression symptoms	ICG	ICG>25	6 and 12 months	6 months: 140 (52.4); 12 months: 112 (54.1)	1	Questionnaire via telephone interview	Logistic Regression; none
**Kentish-Barnes 2017**	30 days after death for relative; unclear for patient	ICG Score and of high risk for CG (Secondary)	ICG	ICG ≥25	6 months	Letter: 38 (27.6); Control: 24 (27.0)	4	Telephone interview	Multivariate logistic regression; stepwise selection (where univariate analysis significant at 5% level or selected for predictive value based on previous reports)
		Primary: HADS score, anxiety and depression							
		Secondary: HADS and IES-R score, anxiety and depression and PTSD							
**Robert 2017 [20]**	At study inclusion	CG (Secondary)	ICG	ICG>25	6 and 12 months	6 months: IE 58 (34.5), TW 80 (43.0); 12 months: IE 55 (36.2), TW 54 (34.0)	1	Clinical data collected	Linear and logistic regression; selected post-hoc based on clinical considerations and between group imbalances
		Primary: PTSD-related symptoms, IES-r score							
		Secondary: patient comfort during dying process, anxiety, depression, job strain score for ICU staff							
**Trevick 2017 [22]**	Majority 1 month after death (some 6 months)	CG (Primary)	ICG-revised, adapted version given to relatives if index patient was still living	ICG-R ≥ 36	1 and 6 months	1 month: 6 (23.1); 6 months: 5 (21.7)	14	Telephone interviews	Mann-Whitney U tests and Spearmen’s correlation; none
		Primary: PTSD; Secondary: IES-r and ICG-r scores							

Abbreviations: CG: Complicated grief; PTSD: Post-traumatic stress disorder; PGD: Prolonged grief disorder; PCBRD: Persistent complex bereavement-related disorder; ICG: Inventory of complicated grief; ICG-R: ICG-revised; HADS: Hospital anxiety and depression scale; IES-R: Impact of event scale (PTSD); CBI-17: Core bereavement items questionnaire; IE: Immediate extubation; TW: Terminal weaning; NA: Not applicable

A total of 61 different risk factors were investigated across all the studies. The risk factors that each study investigated are presented in Table 3. These risk factors were grouped into seven categories, discussed below. A small number of risk factors, mainly demographic ones, were assessed by multiple studies, but most by only a single study.

**Table 3:** Table indicating the risk factors each of the papers investigated and when each risk factor was ascertained/measured.

	Buckley et al 2015	Downar et al 2018	Kentish-Barnes et al 2015	Kentish-Barnes et al 2016	Kentish-Barnes et al 2017 *	Robert et al 2017†	Trevick and Lord 2017
**Demographics/Characteristics**							
**Patient**							
Age	-	-	X	-	X	-	-
**Relative**							
Sex	X	X	X	-	-	-	X
Age	X	X	-	-	-	-	X
Number of people in household	-	-	X	-	X	-	X
College	-	-	-	-	-	-	X
Religious	-	-	-	-	-	-	X
Household income	-	-	-	-	-	-	X
Relationship to deceased	X	-	X	-	X	-	-
**ICU characteristics/variables**							
**Staff**							
Nurse involvement in clinical research	-	-	X	-	-	-	-
Intensivist board certification<2009	-	-	X	-	-	-	-
Nurses >2 years ICU experience	-	-	X	-	-	-	-
**Patients**							
Length of ICU stay	-	-	X	-	-	-	-
Need for vasopressors	-	-	X	-	-	-	-
Died will intubated	-	-	X	-	-	-	-
Immediate extubation vs terminal weaning	-	-	-	-	-	X	-
Refused treatment	-	-	X	-	-	-	-
**Communication Disagreements/Conflict**							
Family disagreement EOL decision	-	-	X	-	-	-	-
Family conflict	-	-	-	-	-	-	X
Team conflict	-	-	-	-	-	-	X
Communication with physician unsatisfactory	-	-	X	-	-	-	-
Communication with nurse unsatisfactory	-	-	X	-	-	-	-
**Family Perceptions/Experiences**							
How prepared for death	X	-	-	-	-	-	-
How drawn out dying process seemed	X	-	-	-	-	-	-
How violent death seemed	X	-	-	-	-	-	-
Extent partner suffered in dying	X	-	-	-	-	-	-
Suffering compared to expectation	X	-	-	-	-	-	-
Opportunity to say goodbye	X	-	X	-	-	-	-
Patients’ dignity not respected	-	-	X	-	-	-	-
Perceived pain	-	-	-	-	-	-	X
Family involved with EOL decision	-	-	X	-	-	-	-
Death not anticipated	-	-	X	-	-	-	-
Present at time of death	-	-	X	-	-	-	-
How often at bedside	-	-	-	-	-	-	X
CAESAR score	-	-	-	X	-	-	-
FS-ICU 24	-	-	-	-	-	-	X
**Other health and social related relative variables**							
Medications for Depression	-	X	-	-	-	-	-
Previous depression diagnosis	-	X	-	-	-	-	-
Symptoms of Depression	-	X	-	-	-	-	-
Symptoms of PTSD	-	X	-	-	-	-	-
Symptoms of Social distress	-	X	-	-	-	-	-
Social support	-	-	-	-	-	-	X
PHQ-9 Score	-	X	-	-	-	-	-
SDI Score	-	X	-	-	-	-	-
IES-r Score	-	X	-	-	-	-	X
**Coping mechanisms/determinants**							
Acceptance	X	-	-	-	-	-	-
Active coping	X	-	-	-	-	-	-
Self-distraction	X	-	-	-	-	-	-
Planning	X	-	-	-	-	-	-
Use of emotional support	X	-	-	-	-	-	-
Religion	X	-	-	-	-	-	-
Positive reframing	X	-	-	-	-	-	-
Seeking social support	X	-	-	-	-	-	-
Self-blame	X	-	-	-	-	-	-
Venting feelings	X	-	-	-	-	-	-
Denial	X	-	-	-	-	-	-
Behavioural disengagement	X	-	-	-	-	-	-
Substance or alcohol use	X	-	-	-	-	-	-
**Other**							
Patient dying or not	-	-	-	-	-	-	X
Receiving a letter	-	-	-	-	X	-	-
BGQ Score	-	X	-	-	-	-	-

Abbreviations: EOL: End of life, FS-ICU 24: Family Satisfaction-ICU 24, SDI: Social Difficulties Inventory (assesses everyday problems in cancer patients), IES-R: Impact of Event Scale-Revised (PTSD), BGQ: Brief Grief Questionnaire (designed to screen for CG), PHQ-9: Patient Health Questionnaire-9 (Depression)

* only reported multivariable results, multivariable model only included variables which were significant at 5% level or were selected for their predictive value based on previous reports, unclear which variables were investigated in univariable analyses

† adjusted for the following variables although point estimates not reported for them: Patient characteristics (age, Knaus score, previous ICU stay during current hospital stay, diagnosis at ICU admission, ICU stay before withdrawal decision, SOFA score, RASS score, FiO2, people in patients room at immediate extubation/first change in ventilator setting for terminal weaning, symptomatic bronchial obstruction or gasping, administration of opioids, hypnotic drugs, or neuromuscular blocking agents, behavioural pain scale score during dying process, time to death from immediate extubation or the first change in ventilator settings for terminal weaning), relative characteristics (age, gender, work status, religion, presence in ICU when patient died)

### Quality appraisal and risk of bias

The results of the quality appraisal are displayed in Table 4. The overall quality of the studies varied. Four could be considered of higher quality, scoring 8 or 9 out of 9. The remaining three were of lower quality.

**Table 4:** Quality appraisal scores using the Newcastle Ottawa scale for included studies.

	Selection				Comparability*	Outcome			Score/9
	Representative of Exposed Cohort	Selection Non-Exposed	Ascertainment of Exposure	Demonstration outcome not present at start	Comparability based on design or analysis	Assessment of outcome	Follow-up Long enough	Adequacy of follow-up	
Buckley et al 2015	1	1	1	1	1	1	1	1	8
Downar et al 2018	1	1	1	1	0	1	1	0	6
Kentish-Barnes et al 2015	1	1	1	1	2	1	1	1	9
Kentish-Barnes et al 2016	1	1	1	1	0	1	1	0	6
Kentish-Barnes et al 2017	1	1	1	1	2	1	1	0	8
Robert et al 2017	1	1	1	1	2	1	1	1	9
Trevick and Lord 2017	0	1	1	1	0	0	1	0	4

* a maximum of 2 can be awarded for this category

### Study results

Table 5 summarises the results by showing significant results at any time point for a risk factor associated with complicated grief and the direction of association, or no effect for univariable and multivariable analyses. Detailed results are available in the Supplement (S1 Table).

**Table 5:** Table indicating whether a risk factor showed a significant association with complicated grief for any time point in the study. Association shown for univariable analysis, multivariable analysis and for other associations, if investigated.

Risk Factor	Univariable analysis	Multivariable analysis
**Demographics/Characteristics**		
**Patient**		
Older Age	Decreased risk	Decreased risk
**Relative**		
Sex (Female)	Increased risk (1)	Increased risk
	No effect (2)	
	Decreased risk (1)	
Older Age	Decreased risk (2)	
	No effect (2)	
Number of people in household	Increased risk (alone) (1)	Increased risk (alone) (2)
	No effect (1)	
College	No effect	
Religious	No effect	
Household Income	No effect	
Relationship to deceased	Increased risk (spouse)(1)	Increased risk (spouse/partner)
	No effect (1)	
**ICU characteristics/variables**		
**Staff**		
Nurse involvement in clinical research	No effect	No effect
Intensivist board certification < 2009	Increased risk	Increased risk
Nurses > 2 years ICU experience	No effect	
**Patients**		
Length of ICU stay	No effect	No effect
Need for vasopressors	Increased risk	
Died while intubated	Increased risk	Increased risk
Immediate extubation vs terminal weaning	No effect	No effect
Refused Treatment	Decreased risk	Decreased risk
**Communication Disagreements/Conflict**		
Family disagreement EOL decision	Increased risk	
Family conflict	No effect	
Team conflict	No result available	
Communication with physician unsatisfactory	Increased risk	Increased risk
Communication with nurse unsatisfactory	Increased risk	No effect
**Family perceptions/experiences**		
How prepared for partner/child death	Decreased risk	Decreased risk
How drawn out dying process seemed	Increased risk	No effect
How violent death seemed	Increased risk	No effect
To what extent they thought partner/child suffered in dying	No effect	No effect
How much partner/child suffered compared to what they expected	Increased risk	No effect
Opportunity to say goodbye	Increased risk (No) (1)	Increased risk (No)
	No effect (1)	
Patients’ dignity not respected	Increased risk	
Perceived pain	No effect	
Family involved with EOL decision	Decreased risk	
Death not anticipated	Increased risk	
Present at time of death	Increased risk	Increased risk
How often at bedside	No effect	
CAESAR score	Increased risk (Low score)	
FS-ICU 24	No effect	
**Other health and social related relative variables**		
Medications for depression	No effect	
Previous depression diagnosis	No effect	
Symptoms of depression	Fair concordance	
Symptoms of PTSD	Fair concordance	
Symptoms of social distress	Fair concordance	
Social support	No result available	
PHQ-9 score	Increased Risk	
SDI score	Increased Risk	
IES-r score	Increased Risk (2)	
**Coping mechanisms/determinants**		
Acceptance	Decreased Risk	No effect
Active coping	No effect	
Self-distraction	Increased Risk	No effect
Planning	No effect	
Use of emotional support	Increased Risk	Increased Risk
Religion	No effect	
Positive reframing	No effect	
Seeking social support	Increased Risk	No effect
Self-blame	Increased Risk	Increased Risk
Venting feelings	Increased Risk	No effect
Denial	Increased Risk	Increased Risk
Behavioural disengagement	Increased Risk	No effect
Substance or alcohol use	Increased Risk	No effect
**Other**		
Patient dying or not	No effect	
Receiving a letter	No effect	
BGQ score	Increased Risk	

Yellow indicates the study showed no effect, green indicates the risk factor was associated with a decreased risk of complicated grief and red/orange indicates the study showed an increased risk for complicated grief. Lighter shades of either colour indicates that there is also a study which shows no effect for the association. Combined green and red/orange indicates conflicting evidence. Unless otherwise specified by the number of studies in brackets, the association was only investigated in a single study. Where applicable, further information is given in brackets relating to the risk factor. Abbreviations: EOL: End of life, FS-ICU 24: Family Satisfaction-ICU 24, SDI: Social Difficulties Inventory (assesses everyday problems in cancer patients), IES-R: Impact of Event Scale-Revised (PTSD), BGQ: Brief Grief Questionnaire (designed to screen for CG), PHQ-9: Patient Health Questionnaire-9 (Depression)

Demographics. Older patient age was associated with a decreased risk in two studies; one conducted univariable analysis (OR 0.98, 95%CI 0.96–0.99; p = 0.017) [6] and the other multivariable (OR 0.95, 95% CI 0.93–0.99, p<0.001) [18]. The gender of the family member had inconsistent associations with complicated grief; in univariable analyses, two studies showed no statistically significant results [17, 21] and the other two had opposing findings with one suggesting females were at an increased risk [6] and the other that men had higher scores on average [22]. Two studies indicated that if the family member was a spouse or partner, or if the individual was living alone, then they were at an increased risk. Two studies indicated that older age of the family member was associated with a decreased risk, although two showed no effect. No other demographic demonstrated an association.

ICU characteristics/variables. The only ICU staff characteristic which showed an association was if the intensivist had been board certified before 2009 (OR 3.82, 95% CI 1.57–3.94; p = 0.003) [6]. Using vasopressors and dying while intubated were both associated with an increased risk. The patient declining ICU treatments was associated with a decreased risk of adverse outcomes among family members.

Communication disagreements/conflict. Family disagreements over end-of life decision-making and problematic communication with the physician or nurse were associated with complicated grief. However, when multivariable analysis was conducted, communication with nurses was no longer a significant factor.

Family perceptions/experiences. Being prepared for the death (beta-coefficient –2.69, 95% CI –3.97, -1.40; p<0.01) [17] and if the family was involved with the end-of-life decision-making (OR 0.57, 95% CI 0.35–0.92; p = 0.022) [6] were the only two factors associated with a decrease in complicated grief. Many of the other factors were associated with an increased risk during univariable analyses but not in multivariable analyses. There was some evidence suggesting not having the opportunity to say goodbye and being present at the time of death was associated with an increased risk.

Other variables related to health and social needs. One study measured the concordance between three health-related questionnaires, measuring depression, post-traumatic stress disorder and social distress and risk of complicated grief. This demonstrated a slight concordance with complicated grief [25], based on the kappa statistic. A previous history of depression (p = 0.09) or use of psychotropic medications (p = 0.74) appeared to have no bearing on the risk for an individual [21].

Coping mechanisms/determinants. One study assessed whether different coping mechanisms in bereavement affected the intensity of an individual’s response [17]. This study indicated that three risk factors were significantly associated with increased bereavement intensity in both univariable and multivariable analysis: denial, self-blame and seeking emotional support. Acceptance of the death was the only coping determinant associated with a decreased risk, although this was no longer statistically significant in multivariable analyses.

## Discussion

This systematic review identified seven studies that assessed 61 potential risk factors for complicated grief in family members bereaved in ICU settings. Despite the wide range of risk factors evaluated in the studies, most were investigated in a single study. Factors associated with a reduced risk of complicated grief included older age of the patient or family member, a patient declining ICU treatments, how prepared the family member was for the death and whether the family member was involved with end-of life care decision-making. Factors associated with an increased risk included living alone, being the spouse/partner of the deceased, the patient dying while intubated, problematic communication with doctors, and being present at time of death. Our review highlights risk factors that may help identify those at highest risk of poor bereavement outcomes. Several of these risk factors are amenable to interventions known to improve the quality of end-of-life care in ICUs, such as high quality communication and involvement of patients and families in end-of-life decision-making. Improved care around death can potentially improve subsequent bereavement experiences.

This study identified several ICU-specific factors not previously noted as risk factors for complicated grief. Where the patient had chosen to decline ICU treatments, family members may well have felt treatments were aligned with patient preferences. In addition, they avoided the potential burdens associated with end-of-life care decision-making when patient wishes were uncertain. In contrast, family members experienced increased risk of complicated grief when a patient died while intubated and receiving mechanical ventilation or if receiving circulatory support with vasopressor therapy. These factors could reflect the impact on family members of the challenges in “negotiating a natural death” in patients receiving invasive life support therapies in a highly technical ICU setting [26]. However, in some cases they may signify family requests for escalating life support therapies when clinicians counsel comfort care, and therefore act as a marker for conflict between family members and clinicians or for family difficulties in accepting death [27].

Communication was another important identified factor. Communication between the patient and family member prior to death has been assessed previously [10, 13, 28], but not communication with the medical staff in the context of bereavement outcomes. Sensitive and effective communication may be even more important in the ICU due to the distressing nature of the circumstances and environment. Problematic communication may make it harder for family members to understand what has happened and thus lead to difficulties during the grieving process. A previous study asking family members to make recommendations for improving end-of-life care identified communication as one of the key issues [29]. The global COVID-19 pandemic has amplified these factors and highlighted their importance [30].

Families who did not have the opportunity to say goodbye to their loved one had increased risk of complicated grief. However, being present at the time of death also increased the risk of complicated grief. This apparent paradox may be explained by the fact that these two factors describe different aspects of end of life care. Withdrawal of life sustaining therapies can lead to clinical signs or respiratory distress, which, if not adequately managed, families might find distressing if present at the time of death [31]. Ensuring that families are prepared for, and supported through, the process of withdrawal of life support may reduce the risk of complicated grief, offering potential targets to improve quality of end of life care.

Evaluation of an individual’s bereavement needs includes understanding coping mechanisms and responses to loss [32, 33]; difficulties in these areas are associated with complicated grief in the non-ICU literature [10, 34]. However, these features were only assessed in a single study in this review, with limited significant associations in multivariable analyses possibly due to the small sample size [17, 35].

Previously published reviews, including those conducted systematically, reported factors associated with complicated grief in non-ICU populations. Two investigated risk factors in family members with caregiving roles [12, 13] and the other two reviews did not specify the population of interest [10, 11]. Several risk factors were identified repeatedly, increasing the strength of the evidence-base. For example, being the decedent’s spouse has been associated consistently with increased likelihood of complicated grief [10–13, 36, 37] and some studies also found parents of the decedent were at an increased risk [11, 13, 36]. Studies identified highly dependent relationships another risk factor [10, 11]. This review identified being a spouse as a risk factor in ICU decedent populations but without details of the spousal relationship. Consistent with previous reviews, we identified younger age of the deceased [10–12] and lack of preparedness for death as being risk factors for complicated grief [10, 12, 13].

A number of factors from previous reviews were non-significant or inconclusive in this review: female relative [10, 11], low income [11], prior psychiatric illness [10–12], violent death [10, 11] and lack of education [11, 12]. This may be because some of these factors were not investigated in specific studies, the small sample size in most studies, or because these factors are of less relative important in the context of the ICU setting.

There are several strengths to this review. Firstly, we searched multiple databases to help identify as many relevant studies as possible. Duplicate screening for study inclusion and quality appraisal aimed to reduce error and bias. In addition, we scrutinised studies to identify those which reported factors associated with complicated grief in secondary analyses, despite the primary aim and study design being unrelated, for example in a randomised controlled trial of a condolence letter [18].

This review had a number of limitations. Studies were restricted to those published in English, potentially leading to eligible studies being missed. Many of the studies has small sample sizes increasing the risk of erroneously reporting no effect due to type II error. Additionally, due to the lack of studies investigating the same risk factors, it was not possible to conduct a meta-analysis.

Similar numbers of deaths occur in critical care and inpatient hospice settings in the UK [38]. Despite the high prevalence of complicated grief and mental illness experienced by families of ICU decedents, pro-active screening and bereavement support are uncommon in ICU settings [39]. Specialist palliative care involvement is and the impact of this on care around death in ICU is unclear. This is in contrast to hospice settings, in which bereavement care is an integral component of holistic care provided to patients and families, before, during and after death. Our findings highlight the need to develop and tailor bereavement follow-up services and screening for risk of complicated grief in the ICU context. Implementing such an approach brings its own challenges. Family member-specific demographics and characteristics are not included routinely in ICU clinical records. Specific risk factors related to perceived quality of communication, conflict and emotional burden may emerge during care but can be difficult to elicit reliably as part of routine care. Despite these challenges, possible models of ICU bereavement support services have been reported [40, 41]. ICU services can learn from other services such as hospices that have already integrated bereavement follow-up care pathways into standard practice, and implement similar models to ensure that families bereaved in ICU are provided with the support that they require. The COVID-19 pandemic with its high ICU death rates has been a catalyst for better palliative care and ICU integration in some areas, with proactive palliative care reviews in ICUs becoming routine [42].

Our review identified risk factors for that are modifiable within the ICU. Improving communication between clinicians and family members, involving families in end-of-life discussions and facilitating opportunities to say goodbye may be targets for interventions [6]. Similarly, patient involvement in decision-making about treatment escalation was associated with a markedly reduced risk of family members developing complicated grief. Whilst this is often impossible at the time of ICU admission due to impaired decision-making capacity, early engagement in advance (anticipatory) care planning with patients and families before a health crisis may have a positive impact on family members, particularly if it helps prepare them for proxy decision-making. Some of these factors have been the subject of clinical trials, with mixed results [43, 44].

## Conclusions

Death is common in ICU settings and bereaved family members are at risk of poor outcomes. Yet, proactive screening and bereavement support are uncommon. This systematic review has identified potentially modifiable risk factors, some of which are specific to the ICU setting, which may help identify family members at highest risk of complicated grief. Our findings highlight the need to develop and tailor bereavement screening and follow-up services for family members bereaved in ICU settings.

## Supporting information

S1 Table Table of results for all risk factors each study investigated along with their corresponding measures of association for each time-point. Results given for both univariable and multivariable analysis, where reported.
(DOCX)

S1 File (DOCX)

S2 File (DOCX)
