# Supplementary material for: Risk factors for complicated grief among family members bereaved in intensive care unit settings: A systematic review
Source: PLoS One. 2022 Mar 10;17(3):e0264971. doi: 10.1371/journal.pone.0264971 (PMC8912194; doi:10.1371/journal.pone.0264971)
Supplement: S1 Table — Results given for both univariable and multivariable analysis, where reported. (DOCX) [file pone.0264971.s001.docx]

# Risk factors for complicated grief among family members bereaved in intensive care unit settings: a systematic review

Emma A M Sanderson [1], Sally Humphreys [2], Fiona Walker [3], Daniel Harris [4], Emma Carduff [5], Joanne McPeake [6], Kirsty Boyd [1], Natalie Pattison [2,7], Nazir I. Lone [1,3]

[1] Usher Institute, University of Edinburgh, Edinburgh, UK

[2] University of Hertfordshire, Hatfield, UK

[3] NHS Lothian, Edinburgh, UK

[4] Cambridge University Hospitals NHS Foundation Trust, UK

[5] Marie Curie Hospice, Glasgow, UK

[6] University of Glasgow, Glasgow, UK

[7] East and North Herts NHS Trust, Hertfordshire, UK

## Medline database search strategy

1. (intensive care unit$ or ICU or Critical care unit$ or "intensive care").ti,ab.
2. critical care/ or intensive care units/ or critical illness/
3. exp bereavement/
4. ((bereav$ or grief or griev$ or mourn$) adj2 (stress$ or distress$ or complicat$ or persist$ or complex or patholog$ or disorder$)).ti,ab.
5. ((bereav$ or grief or griev$ or mourn$) and ("post-traumatic stress disorder" or "PTSD")).ti,ab.
6. exp terminal care/ or exp withholding treatment/
7. palliative care/ or "hospice and palliative care nursing"/ or palliative medicine/
8. ((treatment adj2 withdraw$) or (treatment adj2 withhold$) or (care adj2 withdraw$) or (treatment adj2 withhold$) or "end of life").ti,ab.
9. caregivers/ or family/
10. risk/ or protective factors/ or risk assessment/ or risk factors/
11. (moderat$ or mediat$ or mechan$ or predict$ or caus$ pathway or protective facto$ or facto$ or influence or correlat$ or precurs$ or causal facto$ or risk factor$ or determinant$).ti,ab.
12. 1 or 2
13. (3 or 4 or 5)
14. (6 or 7 or 8) and 9
15. 10 or 11
16. 12 and (13 or 14) and 15
17. (infant or neonat$ or pediatr$ or paediatr$).mp.
18. 16 not 17

**S1 Table**. Table of results for all risk factors each study investigated along with their corresponding measures of association for each time-point. Results given for both univariable and multivariable analysis, where reported.

| **Study** | **Risk Factor** | **Association with outcome** | | | |
| --- | --- | --- | --- | --- | --- |
| Buckley et al 2015 | **Univariate B (95% CI)** | **Univariable: 3m** | **Multivariable: 3m** | **Univariable: 6m** | **Multivariable: 6m** |
|  | Prepared for death | -2.85 (-4.00, -1.69) p<0.001 | p<0.001 | -2.69 (-3.97, -1.40) p<0.001 | p<0.001 |
|  | How drawn out dying seemed | 0.82 (-0.76,2.41) p=0.3 |  | 1.69 (0.01,3.20) p=0.048 | p=0.08 |
|  | How violent death seemed | 1.39 (-0.22, 2.99) p=0.09 | p=0.87 | 2.12 (0.58, 3.65) p=0.007 | p=0.37 |
|  | Extent partner/child seemed to suffer | 1.16 (-0.25, 2.58) p=0.1 |  | 1.35 (-0.03, 2.73) p=0.054 | p=0.64 |
|  | How much partner/child suffered compared to what they expected | 1.24 (-0.05,2.58) p=0.06 | p=0.1 | 1.41 (0.17, 2.65) p=0.03 | p=0.16 |
|  | Opportunity to say goodbye | 3.53 (-2.23,9.31) p=0.26 |  | 1.67 (-4.44, 7.76) p=0.58 |  |
|  | **B (95% CI)** |  |  |  |  |
|  | Acceptance | -2.1 (-3.68, -0.52) p=0.01 | p=0.09 | -1.86 (-3.43, -0.30) p=0.02 | p=0.88 |
|  | Active Coping | -0.85 (-2.79, 1.09) p=0.38 |  | -0.23 (-1.85, 1.39) p=0.77 |  |
|  | Self-distraction | 1.45 (-0.22, 3.12) p=0.08 | p=0.31 | 2.40 (0.86, 3.93) p=0.003 | p=0.06 |
|  | Planning | 0.69 (-0.99, 2.38) p=0.15 |  | 0.92 (-0.56, 2.41) p=0.22 |  |
|  | Use of emotional support | 2.76 (1.14, 4.39) p=0.001 | p=0.26 | 2.15 (0.86, 3.98) p=0.003 | p=0.02 |
|  | Religion | -0.88 (-2.33, 0.58) p=0.23 |  | 0.72 (-0.58, 2.02) p=0.27 |  |
|  | Positive reframing | -0.93 (-2.73, 0.88) p=0.31 |  | -0.20 (-1.73, 1.35) p=0.8 |  |
|  | Seeking social support | 1.74 (0.16, 3.12) p=0.03 | p=0.63 | 1.44 (-0.30, 3.17) p=0.1 |  |
|  | Self-blame | 2.26 (0.13, 4.39) p=0.04 | p=0.59 | 3.30 (1.87, 4.73) p<0.001 | p=0.003 |
|  | Venting feelings | 2.35 (0.17, 4.54) p=0.03 | p=0.49 | 2.50 (0.41, 4.60) p=0.02 | p=0.94 |
|  | Denial | 4.09 (2.57,5.62) p<0.001 | p=0.03 | 4.52 (3.11, 5.92) p=0.001 | p<0.001 |
|  | Behavioural disengagement | 5.58 (2.32, 8.85) p<0.001 | p=0.1 | 3.42 (1.13, 5.71) p=0.004 | p=0.37 |
|  | Substance or alcohol use | 4.15 (0.39, 7.91) p=0.03 | p=0.63 | 1.90 (-0.59, 4.39) p=0.13 |  |
|  | **No specific measure reported** | **3 months** | | **6 months** | |
|  | Men vs Woman | No significant difference | | No significant difference | |
|  | Relationship to deceased | No significant difference | | No significant difference | |
|  | Age | Increased CBI-17 weakly correlated younger age p=0.047 | | No correlation, p=0.23 | |
| Downar et al 2018 | **Adjusted R^2^** |  | | | |
|  | Medications for depression | -4.5 p=0.74 | | | |
|  | Sex | -0.1 p=0.35 | | | |
|  | Previous depression diagnosis | 2.4 p=0.09 | | | |
|  | Age | 3.1 p=0.06 | | | |
|  | BGQ Score>4 | 8.9 p=0.004 | | | |
|  | PHQ Score >9 | 10.2 p=0.002 | | | |
|  | SDI Score >9 | 11.1 p=0.001 | | | |
|  | IES-r Score >32 | 14 p=0.0003 | | | |
|  | SDI Score | 18.9 p<0.0001 | | | |
|  | PHQ-9 Score | 23.5 p<0.0001 |  |  |  |
|  | BGQ Score | 25.7 p<0.0001 | | | |
|  | IES-r Score | 28.1 p<0.0001 | | | |
|  | **Kappa statistic** |  | | | |
|  | Symptoms of depression | 0.28 | | | |
|  | Symptoms of PTSD | 0.30 | | | |
|  | Symptoms of social distress | 0.32 | | | |
| Kentish-Barnes et al 2015 | **Odds Ratio (95% CI)** | **Univariable Results: 6 months** | | **Multivariable Results : 6 months** | |
|  | Nurse involvement in clinical research | 1.57 (0.84-2.92) p=0.155 | | 1.75 (0.84-3.66) p=0.135 | |
|  | Intensivist board certification <2009 | 3.02 (1.38-6.60) p=0.006 | | 3.82 (1.57-9.34) p=0.003 | |
|  | Nurses> 2 year ICU experience | 0.71 (0.41-1.23) p=0.221 | |  | |
|  | Patient age | 0.98 (0.96-0.99) p=0.017 | |  | |
|  | Length of ICU stay | 1.01 (1.00-1.02) p=0.101 | | 1.01 (1.00-1.02) p=0.155 | |
|  | Need for vasopressors | 2.10 (1.20-3.68) p=0.009 | |  | |
|  | Died will intubated | 2.54 (1.53-4.22) p<0.001 | | 2.12 (1.16-3.89) p=0.015 | |
|  | Family involved with EOL decision | 0.57 (0.35-0.92) p=0.022 | |  | |
|  | Family disagreement EOL decision | 3.92 (1.79-8.60) p=0.001 | |  | |
|  | Female sex | 2.87 (1.67-4.94) p<0.001 | | 3.07 (1.62-5.80) p=0.001 | |
|  | Being the spouse | 1.96 (1.19-3.23) p=0.008 | |  | |
|  | Living alone | 1.64 (1.01-2.70) p=0.044 | | 1.97 (1.10-3.51) p=0.022 | |
|  | *As reported by the relative:* |  | |  | |
|  | Patients dignity not respected | 3.18 (1.13-8.82) p=0.028 | |  | |
|  | Death not anticipated | 2.33 (1.18-4.60) p=0.015 | |  | |
|  | Unsatisfactory communication: physician | 3.64 (1.92-6.91) p<0.001 | | 3.27 (1.42-7.50) p=0.005 | |
|  | Unsatisfactory communication: nurse | 3.21 (1.50-6.85) p=0.003 | | 2.26 (0.84-6.10) p=0.106 | |
|  | Patient refused treatment | 0.38 (0.17-0.86) p=0.021 | | 0.24 (0.08-0.69) p=0.008 | |
|  | Did not say goodbye to loved one | 2.28 (1.35-3.85) p=0.002 | | 2.47 (1.30-4.68) p=0.006 | |
|  | Present at time of death | 1.89 (1.18-3.03) p=0.009 | | 2.91 (1.62-5.21) p<0.001 | |
| Kentish-Barnes et al 2016 | **Odds Ratio (95% CI)** | **6 months** | | **12 months** | |
|  | *CAESAR score* |  | |  | |
|  | Low vs Medium | 1.27 (1.10-1.47) p=0.002 | | 1.04 (0.87-1.24) p=0.64 | |
|  | Low vs High | 1.40 (1.20-1.63) p<0.001 | | 1.27 (1.06-1.52) p=0.011 | |
| Kentish-Barnes et al 2017 | **Odds Ratio (95% CI)** | **Multivariable: 6 months** | | | |
|  | Patient age | 0.95 (0.93-0.99)/ year p<0.0001 | | | |
|  | Relative spouse/partner | 3.44 (1.47-8.05) p<0.001 | | | |
|  | Relative lived alone after death | 4.33 (1.81-10.38) p<0.001 | | | |
|  | *No letter vs letter from ICU* | **Univariable: 6 months** | | | |
|  | Median ICG (IQR) | 13 (4-27)/ 16 (8-30),  p=0.07 | | | |
|  | Difference in proportion with CG | 27%/37.6%, p=0.11 | | | |
| Robert et al 2017 | **Odds Ratio (95% CI)** | **Univariable: 6m** | **Multivariable: 6m** | **Univariable: 12m** | **Multivariable: 12m** |
|  | Immediate Extubation vs Terminal Weaning | 0.70 (0.45,1.07) p=0.1 | 0.67 (0.37,1.22) p=0.19 | 1.10 (0.69, 1.76) p=0.68 | 1.07 (0.57-2.02) p=0.83 |
| Trevick and Lord 2017 | **Median ICG (IQR)** | **6 months** | | | |
|  | Relative Gender (Female/Male) | 21 (20-24)/29.5 (26-36.5), p=0.04 | | | |
|  | Household Size (≥3/1-2) | 27 (25-38)/27 (21-34), p=0.44 | | | |
|  | College Education (Complete/Not) | 28 (22-35)/26 (19-34), p=0.40 | | | |
|  | Religiosity (Religious/Not religious) | 28 (26-35)/ 26 (21-34), p=0.70 | | | |
|  | Household income (≥70k/ <70k) | 26 (21.5-31)/38 (34-55), p=0.13 | | | |
|  | Relative Age | -0.432, p=0.045 | | | |
|  | At bedside (Daily/Less) | 28 (23-38)/ 21 (21-27), p=0.16 | | | |
|  | Family conflict (Present/Absent) | 28 (24.5-31.5)/26.5(21.5-34.5), p=0.93 | | | |
|  | Team conflict (Present/None) | 28/26.5 (21-35), p=NA | | | |
|  | Social support (Good/Poor) | 27 (21.5-34.5)/None , p=NA | | | |
|  | Pain (Persistent/Low) | 32.5 (28.5-38.5)/25 (21-28), p=0.16 | | | |
|  | ICU mortality (Died/Survived) | 32.5 (21-35)/ 26 (22-34), p=0.67 | | | |
|  | FS-ICU 24: | 0.247, p=0.27 | | | |
|  | Part 1 | 0.286, p=0.2 | | | |
|  | Part 2 | 0.211, p=0.35 | | | |
|  | **Correlation ICG-R with other tests** | **ICG-R 6 months** | | **ICG-R 1 month** | |
|  | IES-R 1 month | 0.619, p=0.005 | | 0.609, p=0.002 | |
|  | IES-R 6 months | 0.661, p=0.001 | | 0.492, p=0.038 | |
|  | IES-R Highest (of 1 and 6 months) | 0.695, p<0.001 | | 0.618, p=0.001 | |
|  | ICG-R 1 month | 0.633, p=0.005 | | NA | |

Abbreviations: BGQ: Brief Grief Questionnaire (designed to screen for CG), PHQ: Patient Health Questionnaire (Depression), SDI: Social Difficulties Inventory (assessed everyday problems in cancer patients), IES-r: Inventory of Event Scale-Revised (PTSD), FS-ICU: Family Satisfaction-ICU, EOL: end-of-life
